# The Use of CRISPR-Cas9 Genetic Technology in Cardiovascular Disease: A Comprehensive Review of Current Progress and Future Prospective

**DOI:** 10.7759/cureus.57869

**Published:** 2024-04-08

**Authors:** Muhammad Asif, Wahab J Khan, Sadia Aslam, Awais Aslam, Mohammed A Chowdhury

**Affiliations:** 1 Internal Medicine, University of South Dakota, Sioux Falls, USA; 2 Internal Medicine, Essentia Health, Fargo, USA; 3 Cardiovascular Medicine, North Central Heart Institute, Sioux Falls, USA

**Keywords:** healthcare tech, curative, crispr-cas system (clustered regularly interspaced short palindromic repeats)-crispr associated system, future perspective, cardiovascular disease, gene therapy, genetic medicine, mutation

## Abstract

Over the last century, there have been major landmark developments in the field of medicine, enabling us to control and cure various diseases on a larger scale. A few of these include the discovery of antibiotics, the development of vaccines, and the origin of organ and tissue transplants. The continued quest for innovation in microbiology and medicine has helped humankind save millions of lives and decrease morbidity at the global level. Genetic medicine has grown significantly in the last two decades and appears to be the next frontier of curative therapies for chronic diseases. One important landmark in genetic medicine is the development of CRISPR (clustered, regularly interspaced short palindromic repeats) technology. In this article, we describe the basic structure and function of the CRISPR-Cas9 system, which, simply put, consists of an RNA part and a protein. It works as a molecular scissor that can perform targeted cuts followed by repairs in and around the genes of interest to attain favorable translational outcomes. We focused on summarizing recent studies using CRISPR-Cas9 technology in diagnosing and treating cardiovascular disease. These studies are primarily experimental and limited to animal models. However, their results are promising enough to anticipate that this technology will undoubtedly be available in clinical medicine in the coming years. CRISPR-Cas9-mediated gene editing has been used to study and potentially treat congenital heart disease, hyperlipidemias, arrhythmogenic cardiomyopathies, and the prevention of ischemia-reperfusion injury. Despite the current progress, we recognize the several challenges this technology faces, including funding for research, improving precision and reproducible results for human subjects, and establishing protocols for ethical compliance so that it is acceptable to the scientific community and the general public.

## Introduction and background

Medical science has evolved for centuries through novel discoveries resulting in paradigm shifts in diagnosing and treating various diseases, saving millions of lives. A few of them include the illustration of microbial role in disease pathogenesis, which earned Robert Koch a Nobel Prize in medicine in 1905 for his work on tuberculosis. Together with him, the work of Louis Pasteur laid the foundation for principles of vaccination against infectious diseases. In 1912, Alexis Carrel became the first transplant surgeon to be awarded the Nobel Prize for his work on the suturing of vessels and transplantation of organs [1]. Alexander Fleming, with two other scientists, shared the Nobel Prize in Physiology and Medicine in 1945 for their work on penicillin that revolutionized the treatment of infectious diseases.

In 1953, James Watson and Francis Kirk marked another milestone by explaining that the double helix structure of DNA provided the foundation of modern genetics [2]. This led to the exploding interest of scientists in genetic medicine. In 1990, a 4-year-old patient with severe combined immunodeficiency was the first to receive successful genetic treatment involving delivering a healthy ADA gene into her blood cells using a disabled virus [3]. The excitement of medical scientists in discovering the full potential of genetic medicine led to the human genome project in 1992, in which experts from multiple fields worked together to explore the genetic maps of humans and some other selected organisms. They could finally map out 92% of the human genome by 2003. This was supplemented by further studies using advanced technology to document the entire blueprint of the human genome in 2022 [4]. Since the late 20th century, scientists have been working to use the information from the genome study to understand its role in pathogenesis and its potential use in diagnosing and treating disease. After a decade-long quest, two female scientists, Jennifer Duodena and Emanuel Charpentier, discovered a novel technology called CRISPR which enabled us to precisely manipulate the genomic structure to achieve the desired effects [5]. They were co-awarded the Nobel Prize in 2020 in chemistry for this substantial scientific discovery. Therefore, genetic medicine will be the main frontier of innovation in the coming decades. In this review, we highlight the functional principles of CRISPR-Cas9 and the use of this technology in the cardiovascular field. We also enumerate a few significant challenges it faces, advantages over the traditional practice of medicine, and anticipated prospects in detail.

## Review

The basic structure of the CRISPR-Cas9 system

Clustered, regularly interspaced short palindromic repeats (CRISPR) have been described since 2007 as areas of alternating repeat and non-repeat DNA sequences in the bacterial genome that they use to develop immunity against phage viruses [6]. The bacteria would incorporate pieces of the attacking viral DNA into their genome at chromosomal sites bordered by specific genes termed CRISPR-associated (Cas) genes, which were found necessary for this genomic integration [7]. Once integrated into bacterial DNA, this viral DNA PCR helped bacteria develop immunity against similar viruses in the event of future infections. Previously, this integration of external DNA was spontaneous with little or no external control. However, after learning the mechanism of DNA cleavage over the last decade, researchers were able to create numerous forms of chimeric single-stranded guide RNAs with specific base sequences that, along with endonuclease proteins, would help manipulate the target genome not only in bacterial but other eukaryotic cells as well [8].

The CRISPR-associated proteins (Cas9) and guide RNA (gRNA) sequence make up the contemporary CRISPR-Cas9 system. Streptococcus pyogenes (SpCas9) produces the Cas9 protein, the first Cas protein employed in genome editing. Known as a genetic scissor, it is a big DNA endonuclease that cleaves the target DNA to generate a double-stranded break. The nuclease (NUC) lobe and the recognition (REC) lobe are the two sections that makeup Cas-9. The NUC lobe comprises RuvC, HNH, and Protospacer Adjacent Motif (PAM) interaction domains, whereas the REC lobe comprises the REC1 and REC2 domains that bind guide RNA. Each single-stranded DNA strand is cut by the RuvC and HNH domains, whereas the PAM interaction domain gives PAM specificity and starts the binding process to the target DNA [9]. CRISPR RNA (crRNA) and trans-activating CRISPR RNA (tracrRNA) are the two components of guide RNA. While tracer RNA is a lengthy loop that acts as a binding scaffold for the Cas-9 nuclease, crRNA pairs with the target sequence to specify the target DNA. The purpose of guide RNA in prokaryotes is to target viral DNA; however, the gene-editing tool may be synthetically constructed to target nearly any desired gene sequence by combining tracrRNA and crRNA to generate a single guide RNA (sgRNA) [9]. 

How CRISPR-Cas9 works

Three phases are involved in the operation of the CRISPR-Cas9 genome editing tool: 1) Identification of the targeted gene, 2) Cleavage, and 3) Repair. Cas9 is guided to the precise region of the target gene by the specially engineered sgRNA [10]. Double-stranded breaks are produced by the Cas9 nuclease (DSBs). After locating a target site, Cas9 initiates local DNA unzipping, which succeeds in creating an RNA-DNA hybrid. The Cas9 protein is then triggered to cleave DNA as a result. The HNH domain cuts the complementary strand of target DNA, while the RuvC domain cuts the non-complementary strand, resulting in blunt-ended DSBs. Eventually, natural cellular repair processes restore the DSB [11].

**Figure 1:**
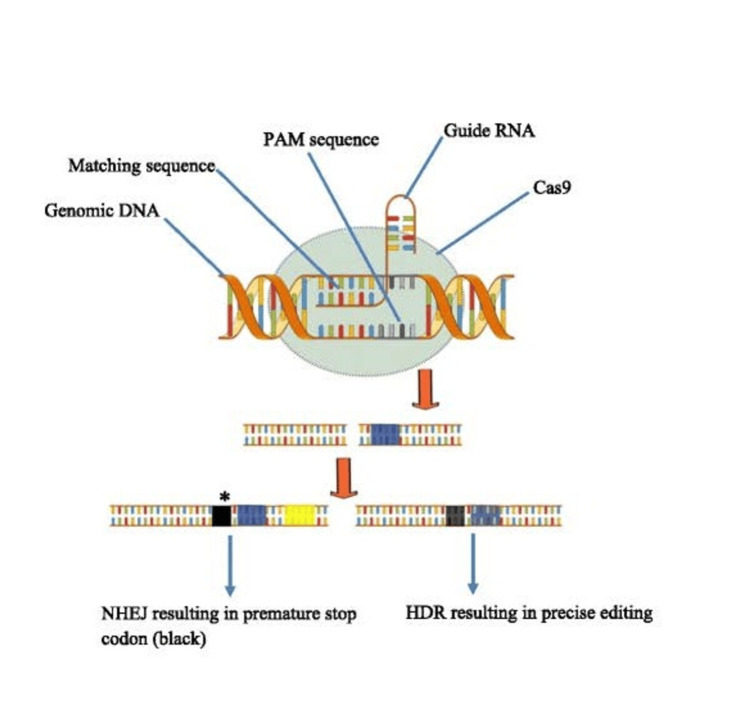
The Mechanism of CRISPR-Cas9 The double-stranded breaks caused by nucleases can be repaired via either homologous end joining or homologous directed repair. (a) NHEJ-mediated repair resulting in gene knockout with no donor DNA. (b) When donor DNA is accessible, it is cut by the nuclease concurrently, resulting in compatible overhangs. Therefore, gene insertion can also occur through NHEJ. (c) When donor DNA is present, HDR enables exact nucleotide substitutions, leading to gene modifications. (d) HDR can also lead to gene insertion. Adapted from Khan et al. published under Creative Commons Attribution 4.0 International License [13]. NHEJ: Non-homologous end joining; HDR: homology-directed repair

**Figure 2:**
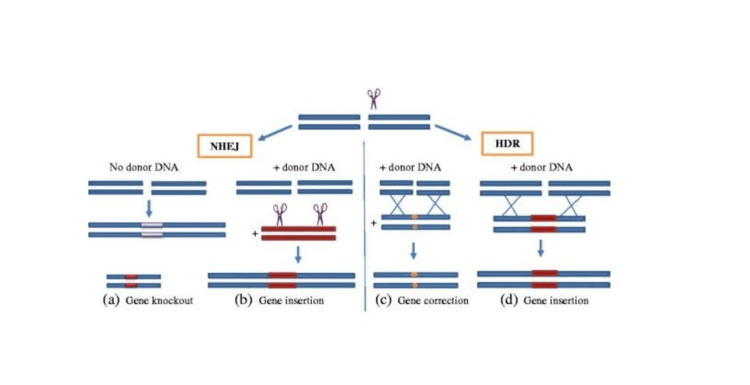
The Mechanisms of CRISPR-Cas9 Cas9 and gRNA are important components of the mechanism. Cas9 operates as a molecular scissors, cutting DNA strands. The gRNA guides Cas9 to break DNA at a specific location. DNA is joined via either NHEJ or HDR. Adapted from Khan et al. published under Creative Commons Attribution 4.0 International License [13]. NHEJ: Non-homologous end joining; HDR: homology-directed repair

 In the CRISPR-Cas9 system, these repair mechanisms can include homology-directed repair (HDR) and non-homologous end joining (NHEJ) to fix double-strand breaks (DSBs) produced by the Cas9 protein (Figures 1, 2). Without exogenous homologous DNA, NHEJ helps repair double-strand breaks (DSBs) by connecting DNA fragments. However, sequencing errors can occur and cause tiny, random insertions or deletions (indels) at the cleavage site, resulting in termination or frameshift mutations (12). While CRISPR gene editing necessitates a substantial quantity of donor (exogenous) DNA templates carrying a sequence of interest, HDR is far more accurate. HDR carries out the exact gene insertion or substitution by introducing a donor DNA template with sequence homology at the anticipated DSB spot. Once we know a specific gene's function and the pathophysiology of the disease of interest, this cycle of targeted cleavage and repair offers the chance and, consequently, the therapeutic basis of a CRISPR-Cas9 system for executing wanted deletion, modification, or gene insertion to obtain desired effects [12].

The impact of CRISPR-Cas9 on diagnosis and management of cardiovascular disease

The Burden of Cardiovascular Disease

Cardiovascular disease (CVD) remains the leading cause of death both in the United States and worldwide. On further segregation, ischemic heart disease is the #1 etiology of deaths attributed to the cardiovascular system [14]. In 2019, cardiovascular disease was the underlying cause of 9.6 million deaths among men and 8.9 million deaths among women, approximately one-third of all the deaths globally [15]. The burden of CVD is measured as the number of disability-adjusted life years (DALY). CVD can be classified into congenital heart disease, ischemic heart disease, valvular heart disease, and cardiomyopathy. With the advancement in medical innovation, treatment outcomes have improved in the last few decades [16]. However, constant improvement in the current practices and use of the latest available technologies for preventative and curative treatment should be the goal of modern medicine. There has been much work done on minimizing the modifiable risk factors, for example, hypertension and smoking, both by chemical treatments and by lifestyle modifications. However, catering to non-modifiable risk factors like familial hyperlipidemia and cardiomyopathies is a significant area yet to be explored. In the following sections, we will review some of the subclasses of cardiovascular disease and highlight the work done so far using CRISPR gene editing technology.

Congenital Heart Disease and Arrhythmias

Congenital heart disease (CHD) is the second most common cause of death after infectious diseases in newborns [17,18]. The overall global burden of this disease, according to the survey in 2017, is 261,247 deaths per annum globally [18]. Rigorous ongoing research on pathogenesis has revealed the various mechanisms as the molecular basis of congenital heart disease. It can be single base mutations, copy number variants, and single nucleotide polymorphisms [19]. CHD is associated with genomic abnormalities combined with environmental factors, the details of which are not yet fully known. The recent development and sequencing technologies have helped scientists reveal underlying genetic perturbations resulting in the phenotypic appearance of CHD. Early on after its inception, the CRISPR-Cas9 in vivo research was focused on mouse models; however, lately, there have been some reviews on strategies for human heart disease. Following are the examples of CHD studied for applying CRISPR-Cas9 technology [20]. Wolf-Parkinson-White syndrome (WPW) is a conduction disorder originating from cardiomyopathy that results in ventricular preexcitation, heart failure, and tachyarrhythmias that can lead to sudden cardiac death [21]. Xie et al. found an H530R mutation in PRKAG2 in patients with familial WPW syndrome. Transgenic and knock-in mice with the H530R PRKAG2 mutation replicated human symptoms of ventricular hypertrophy and glycogen storage, suggesting a causal link to PRKAG2 cardiac syndrome. Adeno-associated virus 9 (AAV9) and the CRISPR-Cas9 gene-editing system were used to remove the mutant PRKAG2 allele that encodes H530R while preserving the wild-type allele. A single systemic injection of AAV9-Cas9/sgRNA at postnatal day four or day 42 successfully restored cardiac structure and activity in H530R PRKAG2 transgenic and knock-in mice. In vivo, CRISPR-Cas9 genome editing can treat dominant hereditary heart disorders, including PRKAG2 cardiac syndrome, by selectively disrupting disease-causing mutations [21].

Duchenne muscular dystrophy (DMD) originates from a mutation in the dystrophin gene that results in more progressive muscle weakness and severe cardiomyopathy. There is no cure for this disease. The MDX mouse model of DMD contains a nonsense mutation in exon 23. Long et al. used a CRISPR-Cas9 strategy to fix this mutation. The investigators administered a Cas9 mRNA/sgRNA/ssODN template for HDR into mice zygotes in the germ line to fix the mutation in each cell. The study examined 11 CRISPR-Cas9 corrected mice, with seven demonstrating effective mutation correction by HDR. This method produced mosaic corrections ranging from 2% to 100%. They found that CRISPR-Cas9-mediated correction in MDX mice reversed the DMD phenotype by histological analysis and serum creatine kinase assays [22].

Holt-Oram syndrome is characterized by upper limb defects and cardiac developmental abnormalities, including septal defects. TBX5 gene, with its 14 potential mutations, is considered a causative gene in this syndrome [23]. Zebrafish with a linear heart tube that fails to loop, mirroring developmental defects in human Holt-Oram syndrome, were studied [24]. Anderson et al. performed a comparative RNA sequencing analysis on zebrafish embryos. Their work showed that knockdown of the TBX5 paralogues results in altered gene expression in many tissues, resulting in changes in somite size, in the differentiation of vasculature progenitors, and later patterning of trunk blood vessels [25].

Heterotaxy syndrome is characterized by left-to-right asymmetry defects affecting the heart. Mutations involving ZIC3 and duplications of the DNAH10 gene located at 12q24.31 have been linked to heterotaxy syndrome. Liu et al. generated CRISPR-Cas9-mediated dnah10 mutants in zebrafish that showed cardiac defects reminiscent of heterotaxy syndrome, thereby providing the first in vivo and functional evidence for dnah10 in left/right patterning. They also expanded the number of causative gene candidates for this syndrome by identifying RNF115 deletion mutations using CRISPR-Cas9 [26]. The therapeutic targeting of these pathogenic genes in the future can lead to exploring therapeutic avenues.

In addition, there have been recent studies of the genetic mechanisms in the pathogenesis of CAKUTHED syndrome in mice [27,28]. Hanses et al. studied Noonan syndrome (NS) using CRISPR Cas9. Their work highlighted the human cardiac pathogenesis in patient-specific induced pluripotent stem cell-derived cardiomyocytes from NS patients carrying biallelic variants in LZTR1 and identified a unique disease-specific proteome signature [29]. They also described the CRISPR repair as a potentially translatable therapeutic strategy to treat NS-associated hypertrophic cardiomyopathy. Watanabe et al. studied DiGeorge syndrome and found that combining the CRISPR-Cas9 system and targeted toxin technology using IB4SAP allows efficient enrichment of genome-edited clones, particularly bi-allelic Knockout (KO) clones [30].

Ischemic Heart Disease, Reperfusion Injury, and Remodeling

The injury incurred to heart muscles from myocardial infarction (MI) happens in various ways: the ischemia itself, the reperfusion injury, and then unfavorable remodeling due to tissue scarring. The last two modalities of myocardial damage are amenable to future treatments by molecular genetic therapy, including the use of CRISPR-Cas9. Abnormal rise in intracellular Ca²⁺ during myocardial reperfusion facilitates cardiomyocyte death and consequent loss of cardiac function, also known as ischemia-reperfusion (IR) injury. Therapeutic modulation of Ca²⁺ handling provides a cardioprotective tool against the paradoxical effects of the definitive therapies of acute ischemia that restore blood flow to the heart. Cardiac IR is also associated with variable changes in microRNA expression, such as miR-214, which is increased during ischemia injury and heart failure. Deleting miR-214 in mice leads to reduced cardiac contractility, increased apoptosis, and excessive fibrosis in response to IR injury [31]. Tang et al. identified miR-150 as a key regulator of cardiomyocyte survival following cardiac damage. Deleting miR-150 in mice results in aberrant cardiac structural and functional remodeling following MI. The miR-150 suppresses pro-apoptotic genes in cardiomyocytes, contributing to its cardioprotective effects during ischemic injury [32]. Both studies reinforced the importance of these micro RNAs as therapeutic targets of future genetic medicine. Lebek et al. showed that editing the CaMKIIδ gene in cardiomyocytes derived from human-induced pluripotent stem cells to eliminate oxidation-sensitive methionine residues confers protection from IR injury [33].

Moreover, CaMKIIδ editing in mice at the time of IR enables the heart to recover function from otherwise severe damage; therefore, CaMKIIδ gene editing may thus represent a permanent and advanced strategy for heart disease therapy [34]. The human heart muscle has limited regenerative potential after myocyte cell death. Nam et al. were successful to some extent in reprogramming human fibroblasts towards cardiomyocytes using four human cardiac transcription factors in laboratory cultures for 4-11 weeks [34]. This provides another therapeutic opportunity to make up for the lost myocardium in ischemic injury.

Hypercholesterolemia

High levels of low-density lipoprotein cholesterol are the third globally attributable modifiable risk factor contributing to the overall burden of cardiovascular disease [14]. Statins, ezetimibe, and proprotein convertase subtilisin/kexin type 9 (PCSK9) inhibitors have been the mainstay treatments to pharmacologically treat hyperlipidemia that nearly always need lifelong treatment, that come along with several potential side effects, putting significant economic and personal burden to the patient and health system overall. Statins mainly act by reducing the production of harmful lipid molecules along with their anti-inflammatory effects. On the other hand, PCSK9 is a regulatory molecule that causes the internalization and degradation of the LDL receptor and thus reduces hepatic LDL uptake. Monoclonal antibodies against PCSK9 increase hepatic LDL-receptor expression, lower circulating LDL-C, and reduce CV events [35]. Recent experimental studies suggest that CRISPR gene-editing targeted at PCSK9 may offer a promising tool to achieve the elusive goal of a ‘once and done’ treatment approach to LDL-C reduction [36]. In an experimental study, Musunuru et al. delivered lipid nanoparticles to precisely modify disease-related genes in living cynomolgus monkeys. They observed a near-complete knockdown of PCSK9 in the liver after a single infusion of lipid nanoparticles; it was accompanied by reductions in blood levels of PCSK9 and LDL cholesterol of approximately 90% and 60%, respectively; moreover, these changes persisted for eight months after the single-dose treatment [37]. Similarly, significant reductions in total cholesterol levels have been seen in mice with targeted mutagenesis efficiency as high as 90%, resulting in a 40% reduction in blood cholesterol levels [38]. It is so evident that treating a wide range of hyperlipidemias with these advanced tools in the future can significantly cut the burden of atherosclerotic CVD.

Cardiomyopathies

Although autosomal recessive, X-linked, and mitochondrial inheritance patterns have also been reported, autosomal dominant disorders are the most common mode of inheritance for hereditary cardiomyopathies, including Duchenne, hypertrophic, and arrhythmogenic cardiomyopathy. These hereditary cardiomyopathies are appealing targets for CRISPR-Cas9 gene editing, as are X-linked Emery-Dreifuss muscular dystrophy (EDMD) and Barth syndrome. In this field, two of the most prevalent single exon deletions that cause DMD have been corrected by scientists in mice. In addition to effectively restoring dystrophin protein expression and maintaining muscle strength and function, systemic and intramuscular administration of AAV9-SpCas9 and AAV9-sgRNA targeted either exon 45 or exon 51 also prevented pathological myofiber degeneration and fibrosis. Liu et al. emphasized using crisper technology to model cardiovascular illness and investigate potential gene therapy therapeutic targets [39].

ABE was used in mouse zygotes in a different study to correct a pathogenic MYH6 R404Q/+ (c1211 C>T) mutation in a mouse model for hereditary HCM. The point mutation was corrected with excellent efficiency by germline injection of ABEmax-NG mRNA and sgRNA base editing components, reducing the amount of mutant mRNA and eliminating HCM in the postnatal mice and their offspring [37]. Additionally, it was demonstrated that ABE might treat a fatal lysosomal storage disease in utero, improving survival and lessening abnormalities in the liver, heart, and musculoskeletal system [38]. These two previously mentioned studies described highly accurate and efficient repair of the mutations in mice. Phospholamban is a transmembrane protein of the sarcoplasmic reticulum. Sarcoplasmic/endoplasmic reticulum calcium ATPase 2 activity is inhibited by this protein, which controls the management of calcium [40,41]. Cardiomyopathies that are dilated, hypertrophic, or arrhythmic have been linked to PLN mutations. ACM and dilated cardiomyopathy have been linked to a 3 bp deletion in phospholamban that results in the loss of R14 [42,43]. One potential target for future CRISPR-Cas9-mediated gene therapy is the PNL gene, among many others that contribute to hereditary cardiomyopathies.

Heart Transplant

Even when cardiovascular patients receive the greatest therapy available, advanced refractory heart failure is an inevitable endpoint for many of them. For many patients, a heart transplant is still their last but only option; yet, demand and supply have never matched, and a sizable portion of patients pass away while waiting for an organ. The current focus of research is on creating organs from stem cells or induced pluripotent stem cells (iPSCs) and altering genomes of donor organ individual cells to increase the graft's tolerance to injury and suitability for allo- and xenotransplantation. Research on CRISPR gene editing in conjunction with pluripotent stem cells (PSCs) and induced stem cells (iPSC) has created enormous opportunities to learn about regenerative medicine and disease contexts [44]. The research focuses on pigs; however, there are three significant obstacles standing in the way of effective organ transplantation from pigs to humans: i) Porcine endogenous retroviruses (PERV) in the porcine genome can transfer vertically into human cells and cause xenosis; ii) dysregulated coagulation due to the difference between the pig and human coagulation system (THBD, TFPI, CD39); and iii) glycans on the surface of porcine endothelial cells act like xenoantigens (α-Gal, Neu5Gc, SDa) and cause hyperacute rejection (HAR). Using CRISPR technology, G. Church and colleagues created the most sophisticated transgenic pig ever. They were able to inactivate 25 PERV loci in pig cells and successfully delete three pig genes (GGTA1, CMAH, B4GALNT2) in addition to specifically inserting nine human transgenes (CD46, CD55, CD59, B2M, HLA-E, CD47, THBD, TFB1, and CD39) in a single locus [45]. Notably, genome project (GP)-write, which aims to write a virus-free genome with the appropriate alterations, would probably be more feasible with the use of CRISPR technology and future devices [44].

Theoretically, infusing human PSCs into an animal blastocyst (blastocyst complementation) or a targeted organ in utero (utero transplantation) can produce patient-specific and immune-matched chimeric organs [46]. Considering the aforementioned developments, it is now not beyond imagination that in the future, we may not remain limited to the availability of donor organs for transplant.

Challenges to clinical use of CRISPR-Cas9 technology

In the last decade, the research in genomic editing has advanced significantly. However, many challenges must be overcome before it can be used clinically in humans. A few of the technical challenges that lie ahead include the precision of targeting, the inability to reproduce the same results, off-target site DNA breaks, and repair in an unwanted fashion that can lead to undesired gene creation and pathologic expression. By creating more accurate and targeted cas9 variants, enhancing the specificity of sgRNA, potentially shrinking the size of Cas9, and experimenting with modified carrier viruses, the target and intended repair are being made précised. However, predicting a similar genetic edit and transitional alteration will still be challenging when moving from an experimental model to a clinical one in a different species [36,39]. Delivering the modified gene from an in vitro editing device for in vivo therapy presents another challenge. Different editing levels, partially or fully modified organs, may be required; however, the existing distribution system does not support these needs. Furthermore, it has been demonstrated that more than 60% of people already have an adaptive humoral and cell-mediated response against the main CRISPR system molecules, such as Cas9 [47]. Consequently, before using Cas9 protein more safely in clinical settings, consideration must be given to its immune response.

The funding of research, the patent rights of technology, high cost, and low usage due to low disease incidence, for example, congenital heart disease, are a few of the commercial challenges this tech industry will have to overcome. Another significant barrier lying ahead of gene editing is the ethical dilemma. Every country and nation have variable beliefs and may have different approaches toward somatic changes in the human species that would originate from gene therapy [48]. There is a concern about whether this therapy can be used one day to enhance or favorably alter various human characteristics such as appearance, learning ability, memory function, or endurance tolerance. Moreover, if so, will this change be equitable to all human beings or only a set of resourceful people putting others on low ground? Will it be used to create genetic copies of humans with desired traits? For example, an Olympic athlete or a supermodel. While changing somatic cells may affect one individual, a genetic change introduced in human germline cells can affect many generations. Therefore, it is the subject of considerable debate about the best oversight and regulatory mechanisms that can ensure the safe clinical use of this technology while avoiding any malicious or unintentional harm to human life. In 2019, scientists from various countries recommended a moratorium on human germline editing until safety and acceptable ethical principles are defined and regulatory mechanisms are ensured [49].

## Conclusions

CRISPR-Cas9 is a promising gene editing technology used to study the genetic basis of diseases, including those of the cardiovascular system, and achieve cure by purposefully altering relevant genetic material. However, there are various challenges to its being at very primitive stages, involving knowledge of genetic pathophysiology of the disease, precision of gene targeting and repair, high cost and low usage on the community level, and ethical considerations to oversee its usage. Nonetheless, its successful use in the coming years may completely change the outlook of medical practice, including CVD.
